# Long-term follow-up of 109 children with juvenile idiopathic oligoarthritis after first intra-articular corticosteroid injection

**DOI:** 10.1186/s13075-024-03303-y

**Published:** 2024-03-14

**Authors:** Mojca Zajc Avramovič, Nataša Toplak, Gašper Markelj, Nina Emeršič, Tadej Avčin

**Affiliations:** 1 Department of Allergology, Rheumatology and Clinical Immunology, Children’s Hospital, University Medical Centre Ljubljana, Bohoričeva ulica 20, Ljubljana, 1000 Slovenia; 2 Department of Pediatrics, Faculty of Medicine, University of Ljubljana, Ljubljana, Slovenia

**Keywords:** Juvenile idiopathic arthitis, Oligoarthritis, Pediatric arthritis, Outcome, Long-term follow-up, ANA, HLA B27, Biologic therapy, Methotrexate

## Abstract

**Background:**

To evaluate long-term outcomes and prognostic factors in patients with juvenile idiopathic arthritis (JIA), presenting as oligoarthritis, who received IAC as the first treatment for their disease.

**Methods:**

We conducted retrospective study at the University Children’s Hospital Ljubljana, Slovenia, from January 2015 to May 2023 in children with JIA, clinically presenting as oligoarthritis receiving intra-articular corticosteroid injection (IAC) as the initial treatment. Patient and treatment data were collected, and the outcomes were categorized into three groups based on the later need for therapy: no therapy needed, only additional IAC needed and systemic therapy needed. The last group was further divided based on the requirement of bDMARD. Log-rank (Mantel-Cox) survival analyses compared different outcome groups.

**Results:**

We included 109 patients with JIA, presenting as oligoarthritis (63% female), who were first treated with IAC. The mean age at IAC was 8.0 years, with a 4.3-year follow-up. Notably, 38.5% of patients did not require additional therapy post-IAC, whereas 15.5% required only additional IAC. Systemic therapy, mainly methotrexate (MTX), was necessary for 45.9% of patients, initiated in average 7.8 months post-IAC. Biologic therapy was initiated in 22% in average 2.2 years post-IAC. Number of injected joints correlated with the need for biologics. At the last follow-up, 88.9% had inactive disease. ANA positivity (*P* = 0.049, chi square 3.89) and HLA B27 antigen presence (*P* = 0.050, chi square 3.85) were associated with the need for systemic therapy. A subgroup of children older than 8 years, ANA and HLA B27 negative required significantly less systemic (25.8%) and biologic therapy (9.6%) compared to other patients (*p* = 0.050, chi square 3.77).

**Conclusion:**

Almost 40% of children with oligoarticular JIA requiring IAC did not progress to chronic disease. Younger age, ANA positivity, and HLA B27 presence were predictive factors for systemic therapy, while the number of injected joints predicted the future need for biologic therapy.

## Background

Juvenile idiopathic arthritis (JIA) is an umbrella term that encompasses all idiopathic arthritides in children lasting at least six weeks with excluded other causes [1]. It is one of the most common chronic diseases in childhood with estimated global incidence rates of 1.6 to 23 per 100,000 children and estimated global prevalence rates of 3.8 to 400 per 100,000 children [2]. In Europe, the estimated annual number of incident cases in 2010 was 6896 with 59,175 prevalent cases [3]. It is classified into 7 categories, according to the revised International League of Associations for Rheumatology (ILAR) criteria [4]. Oligoarticular JIA is the most common category encompassing 27–56% of JIA [1]. Itis further divided into the persistent oligoarticular form, where the number of involved joints remains ≤ 4, or the extended oligoarticular form, where at least five joints are involved in the course of the disease [4]. The ILAR classification is not completely satisfactory, and with time, patients can often present a different subtype with onset of new symptoms, whilst others remain undifferentiated for years [5]. In the treatment approach American College of Rheumatology (ACR) guidelines oligoarthritis refers to JIA presenting with involvement of ≤ 4 joints without systemic manifestations [6]. It may include patients with different categories of JIA who share a common limited number of joints involved. In the present study the term oligoarthritis is used in line with ACR definition as treatment approach was used to select patients.

Intra-articular glucocorticoids (IAC) are strongly recommended as part of the initial therapy for active oligoarthritis [6]. Some patients achieve sustained remission after first IAC and do not develop chronic disease on long-term. Some children later progress and develop a chronic disease that requires conventional disease modifying antirheumatic drugs (cDMARDs) and/or biologic disease modifying antirheumatic drugs (bDMARDs). When a patient is seen in the first months from the disease onset, it is very difficult to predict the outcome. It is an unanswered question among parents and physicians alike how to predict different course and prognosis of these patients at the start of the disease.

## Objective

To evaluate the long-term outcomes of patients with JIA oligoarthritis who received IAC as the first treatment for their disease. In addition, the parameters influencing the different outcomes were investigated.

## Methods

### Study design and patient selection

This was a single-center, retrospective study with longitudinal follow-up. We enrolled consecutive children with idiopathic oligoarthritis lasting at least six weeks, whose first treatment for arthritis was IAC. The study was conducted at the University Children’s Hospital Ljubljana (UCHL), Slovenia. Patients that received IAC from January 2015 to May 2020 were enrolled, allowing the minimum follow-up of 3 years. Patient data was obtained through the UCHL Registry of children with immune-mediated disorders, from the JIA sub-registry. The registry has been approved by the National Ethics Committee for Research in Medicine with the reference number 0120–536/2020/3. Written consent was obtained from all patients and their parents/legal guardians. Data were reviewed until last follow-up visits. Nonsteroidal anti-inflammatory drugs were allowed before and at the time of IAC. An exclusion criterion was systemic immunomodulatory therapy at the time of or in the first 30 days after IAC. For further analyses we excluded also patients with associated conditions that could at least partially be affecting the arthritis.

### Data collection

Collected data included demographic data, JIA category, antinuclear antibody positivity (ANA; in the titer 1:160 or higher), presence of HLA B27 antigen, number of affected joints, uveitis anytime in the course of the disease, treatment data and disease activity at last follow-up. Disease activity was measured using Wallace criteria. Inactive disease was defined as absence of joints with active arthritis, uveitis, enthesitis, normal inflammatory markers and physician global assessment indicating no disease activity (visual analogue scale = 0). Remission while taking therapy was defined as continuously inactive disease for 6 months, and remission off therapy as continuously inactive disease for 12 months without any antiarthritis drug [7]. 

### Analysis

Regarding the outcome, patients were divided into 3 groups: (1) patients, that after first IAC required no further therapy, (2) patients, that after first IAC required only additional IAC but no systemic therapy, (3) patients, that required systemic therapy with cDMARD. The third group was further divided into two subgroups: (3a) patients, who required only cDMARD, (3b) patients, that required bDMARD.

All results are expressed as mean ± SD or median (range). Mann-Whitney U test, Chi square, two way ANOVA, and Fisher exact test were used as appropriate. The following data were considered as variables and as covariates for the survival curves: age at IAC, sex, ANA positivity, HLA B27 presence, time to additional IAC, time to MTX, time to biological therapy. Log-rank (Mantel-Cox) survival analyses were performed to compare groups with different outcomes. P values less than 0.05 were considered statistically significant. Data was analysed and visualized using GraphPad Prism 9 software and SankeyMATIC.

## Results

116 patients were enrolled. We excluded 7 patients (4 that had suspected B.burgdorferi infection prior to arthritis, 1 boy with hondromalatiae patelae, 1 girl with parapatellar plica and 1 boy, who two years after arthritis developed ulcerous collitis. For further analysis the cohort included 109 patients, 69 (63.3%) were female. Study group characteristics are presented in Table 1. Triamcinolone hexacetonide was used in all patients. The average age at the time of first IAC was 8.0 years (1.2–18.3). All patients were white. Regarding the ILAR classification, 73% of patients had persistent oligoarticular JIA, 11% had extended oligoarticular JIA, 5% had psoriatic arthritis and 10% had enthesitis related arthritis.

**Table 1:** Characteristics of groups

	Study group	No further therapy	Only additional IAC	Systemic therapy	Biologic therapy
Number % *(n)*	109	38.5% *(42)*	15.5% [17]	45.9% (*49* MTX, *1* SSZ)	22.0% *(24)*
Age (years)	8.0 (1.2–18.3)	9.2 (1.2–18)	6.9 (1.5–13.5)	7.1 (1.5–18.3)	7.7 years (1.8–18)
ANA +	38% *(41)*	26.8% (11)	29.5% (5)	48% *(24)*	54% (13)
HLA B27 +	14% (15)	7.5% (3)	17.6% (3)	18% (9)	25% (6)
First IAC: - 1 joint - 2 joints - 3 joints	76% *(83)* 22% *(24)* 2% (2)	83% *(35)* 14.2% (6) 2% (1)	70.6% (12) 39.4% (5) /	74%*(36)* 24% (12) 2% (1)	54% (13) 42% (10) 4% (1)
Mean follow-up	4.3 yrs (7 mo – 8.2 yrs)	3.3 yrs (7 mo – 7.8 yrs)	4.7 yrs (1.4–6.7)	5.1 yrs (1.4–8.1)	5.2 yrs (1.4–8.1)
Inactive disease at last follow-up visit	88.9% (97)	100%	100%	74% (36)	75% (18)
Remission off therapy at last follow-up visit	70.6% (77)	100%	100%	44% [16]	11% [2]

**Fig. 1:**
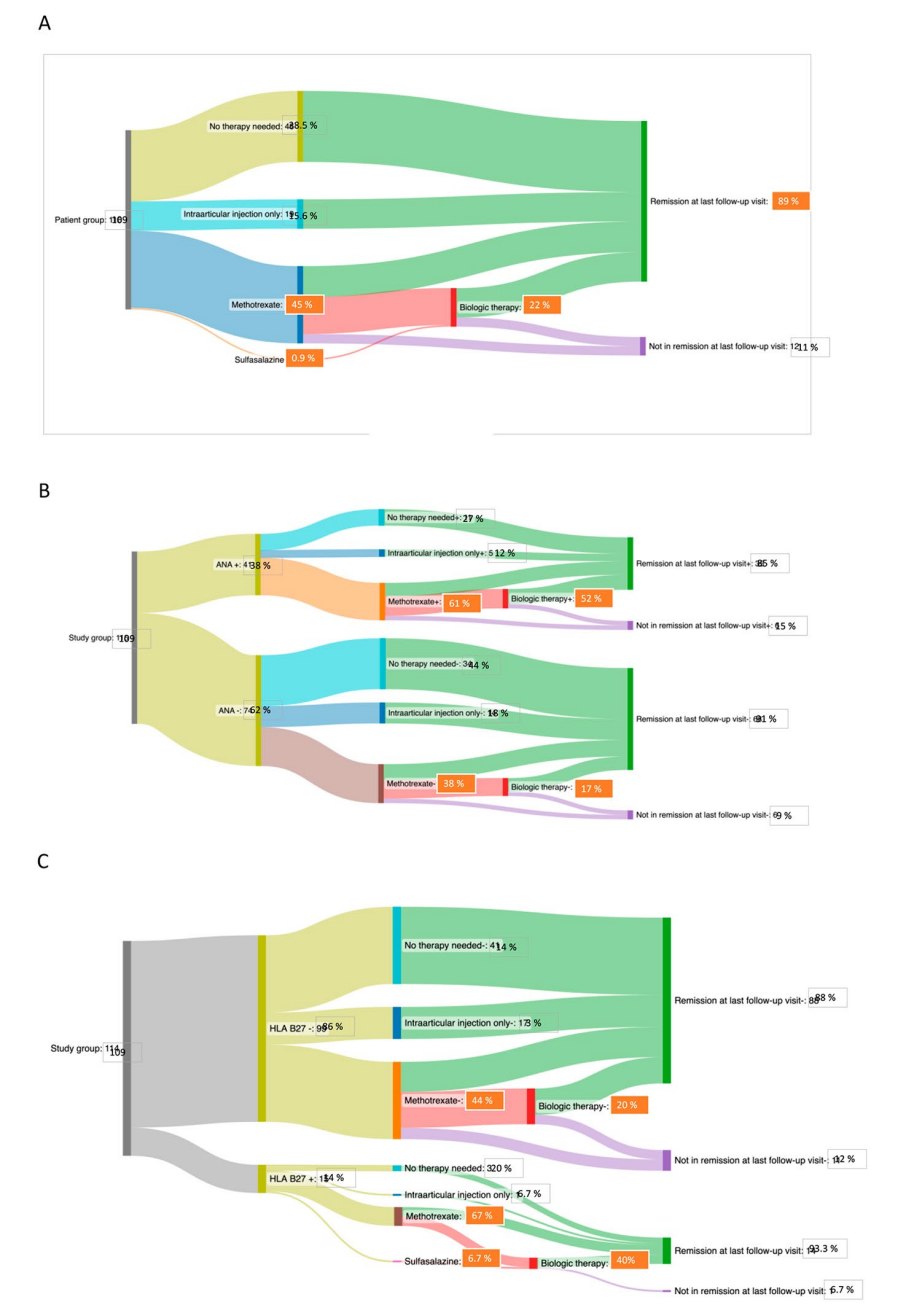
The course of disease and therapy in study group and subgroups. **A**. The course of disease and therapy in the whole study group after first intra-articular corticosteroid injection. **B**. Disease course and therapy after first intra-articular corticosteroid injection. Patients are divided based on presence of ANA. **C**. Disease course and therapy after first intra-articular corticosteroid injection. Patients are divided based on the presence of HLA B27 antigen

After the first IAC 38.5% (42/109) did not require any further therapy and 14.7% (16/109) only required additional IAC. Systemic therapy was needed during the follow up in 45.9% (50/109) of patients, with 49 receiving methotrexate (MTX) and 1 receiving sulfasalazine (SSZ). Biologic therapy was introduced in 22.0% (24/109) of patients. At the last follow up visit 88.9% (97/109) had inactive disease. The patients were followed for the mean time 4.3 years (7 months – 8.2 years). Trajectory of treatment after first IAC is shown in Fig. 1. Regarding immunoserology 38.0% (41/108; one patient had missing data) were ANA positive, 14.0% (15/107; 2 patients had missing data) were HLA B27 positive, all patients were RF negative. In 76.1% (83/109) of children one joint was injected, in 22.0% (24/109) two joints and in 2 patients 3 joints were injected at the time of first IAC.

### The ILAR categories

**Fig. 2:**
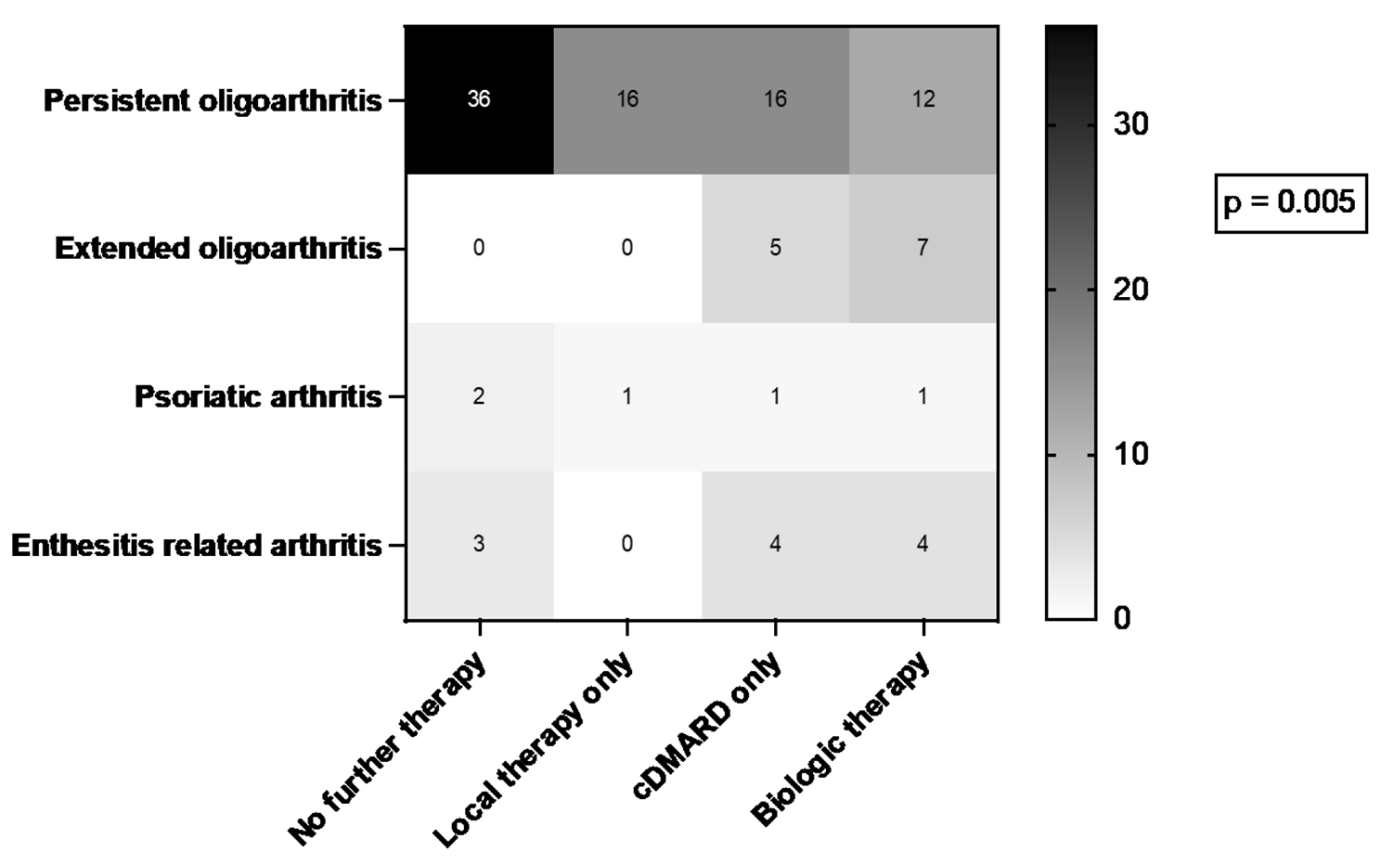
Therapy regarding the ILAR category. ILAR category was significantly associated with therapy requirement using two-way ANOVA (*p* = 0.005). Numbers of patients are shown in each cell

The ILAR category was significantly associated with the required therapy (*p* = 0.005). Results are shown in Fig. 2. No further therapy was required in 45% of patients with persistant oligoarticular JIA, in no patient with extended oligoarticular JIA, in 40% of patients with psoriatic arthritis and 27.3% of patients with enthesitis related arthritis (ERA). Further local therapy was required in 20% of patients with persistant oligoarticular JIA, in no patient with extended oligoarticular JIA, in 20% of patients with psoriatic arthritis and in no patients with ERA. Systemic therapy was required in 35% of patients with persistant oligoarticular JIA, in all patients with extended oligoarticular JIA, in 40% of patients with psoriatic arthritis and in 72.3% patients with ERA. Biologic therapy was required in 15% of patients with persistant oligoarticular JIA, in 58% patients with extended oligoarticular JIA, in 20% of patients with psoriatic arthritis and in 36.4% patients with ERA.

### No further therapy required

In this group of 42 children the mean age was 9.2 (1.2–18) years, which was older than in the group that later required systemic therapy but the difference was not statistically significant (*p* > 0,05). 26.8% (11/41, one patient missing data) were ANA positive and 7.5% (3/40, 2 patients missing data) were HLA B27 positive. In 83.3% (35/42) only one joint was involved, in 14.2% (6/42) two joints were involved and one patient (2.3%, 1/42) had 3 joints injected. They were followed by mean of 3.3 years (7 months – 7.8 years). All patients were in remission off therapy at the last follow-up visit.

### Only additional IAC required

In this group mean age was 6.9 years (1.5–13.5), 29.5% (5/17) were ANA positive, 17.6% (3/17) were HLA B27 positive. The number of affected joints was one in 70.6% (12/17) and 2 in 39.4% (5/17) of patients. In average, patients required additional IAC after 1.9 years (4 months – 6 years). They were followed by mean of 4.7 years (1.4–6.7). All patients were in remission off therapy at the last follow-up visit.

### Disease modifying anti-rheumatic drugs (DMARDs)

Systemic therapy was required in 45.9% (50/109) of patients, with 49 receiving MTX and 1 receiving SSZ. We only included patients receiving MTX in further analyses and among those the average age at disease onset was 7.1 (1.5–18.3) years, 48.9% (24/49) were ANA positive, 18.4% (9/49) were HLA B27 positive. Only one joint was injected at first IAC in 73.5% (36/49), two joints in 24.4% (12/49) and three joints in 2% (1/49). Number of injected joints was not associated with requirement for systemic therapy with MTX (*p* = 0.5). Five patients (10.2%) developed uveitis in the course of the disease. MTX was introduced median 7.1 months (mean 14.2 months; 1 month − 6.1 years) after first IAC. The mean follow up was 5.1 years (1.4–8.1). During the follow-up 53.1% (26/49) had methotrexate as the only systemic therapy. At the last follow-up visit 73.5% (36/49) had inactive disease and 44% (16/36) of them were off therapy.

### Biologic therapy

**Fig. 3:**
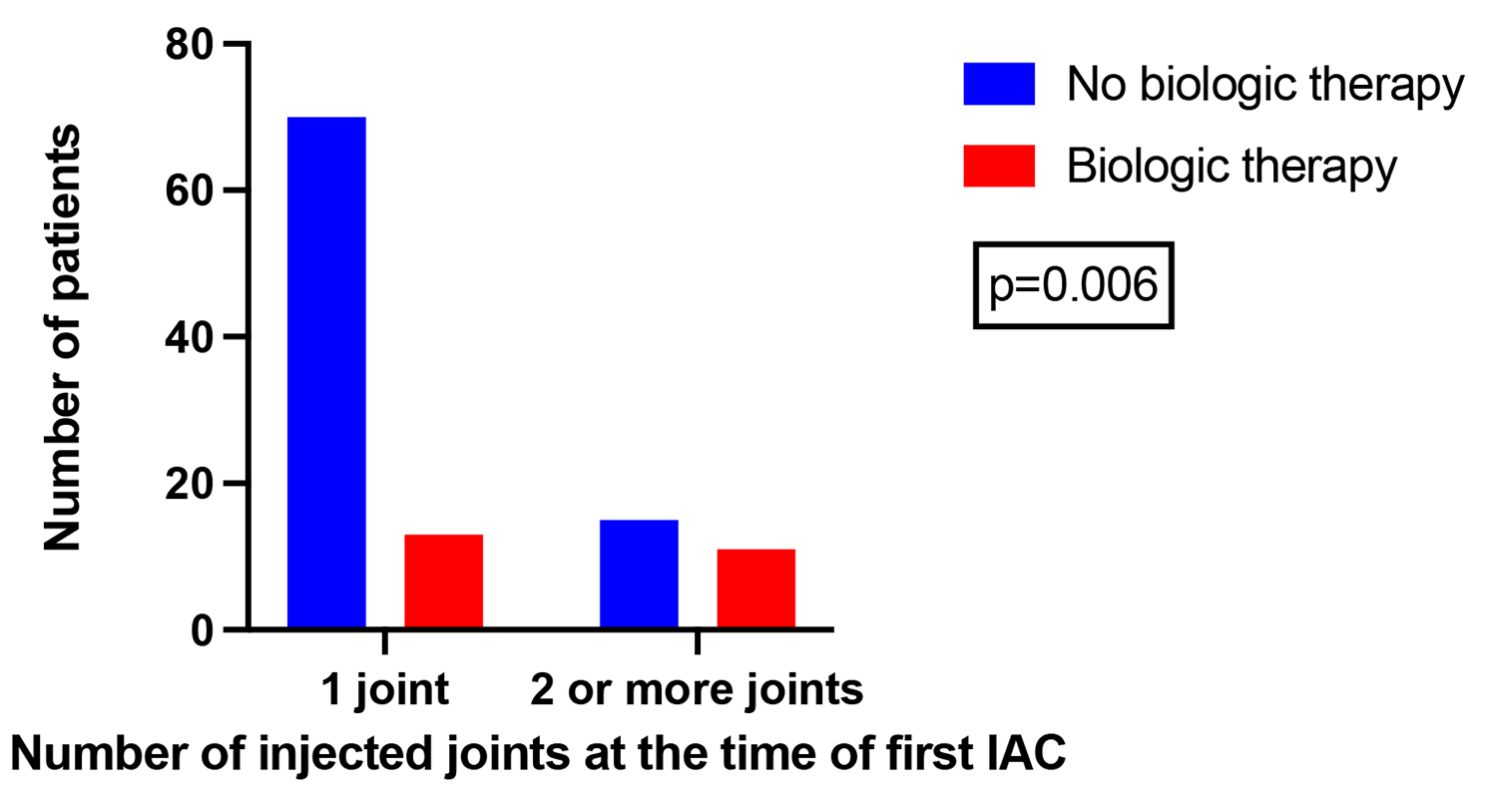
Association of the number of injected joints at first intra-articular corticosteroid injection with the requirement for biologic therapy. Association is statistically significant using Fisher exact test

22% (24/109, 23 receiving MTX, 1 receiving SSZ) were eventually treated with biologic therapy. The mean age in this group was 7.7 years (1.8–18), 54.2% (13/24) were ANA positive, 25% (6/24) were HLA B27 positive. At the time of first IAC one joint was involved in 54.2% (13/24), two joints in 41.7% (10/24) and 3 joints in 4.2% (1/24) of patients. The number of injected joints was significantly associated to the requirement for biologic therapy using Fisher exact test (*p* = 0.006), presented in Fig. 3. They were followed for 5.2 years in average (1.4–8.1) and the mean time to biologic therapy was 2.2 years (3 months – 4.6 years). At the last follow up visit 75% (18/24) had inactive disease, 11.1% (2/18) of them were off therapy.

### ANA positivity

**Fig. 4:**
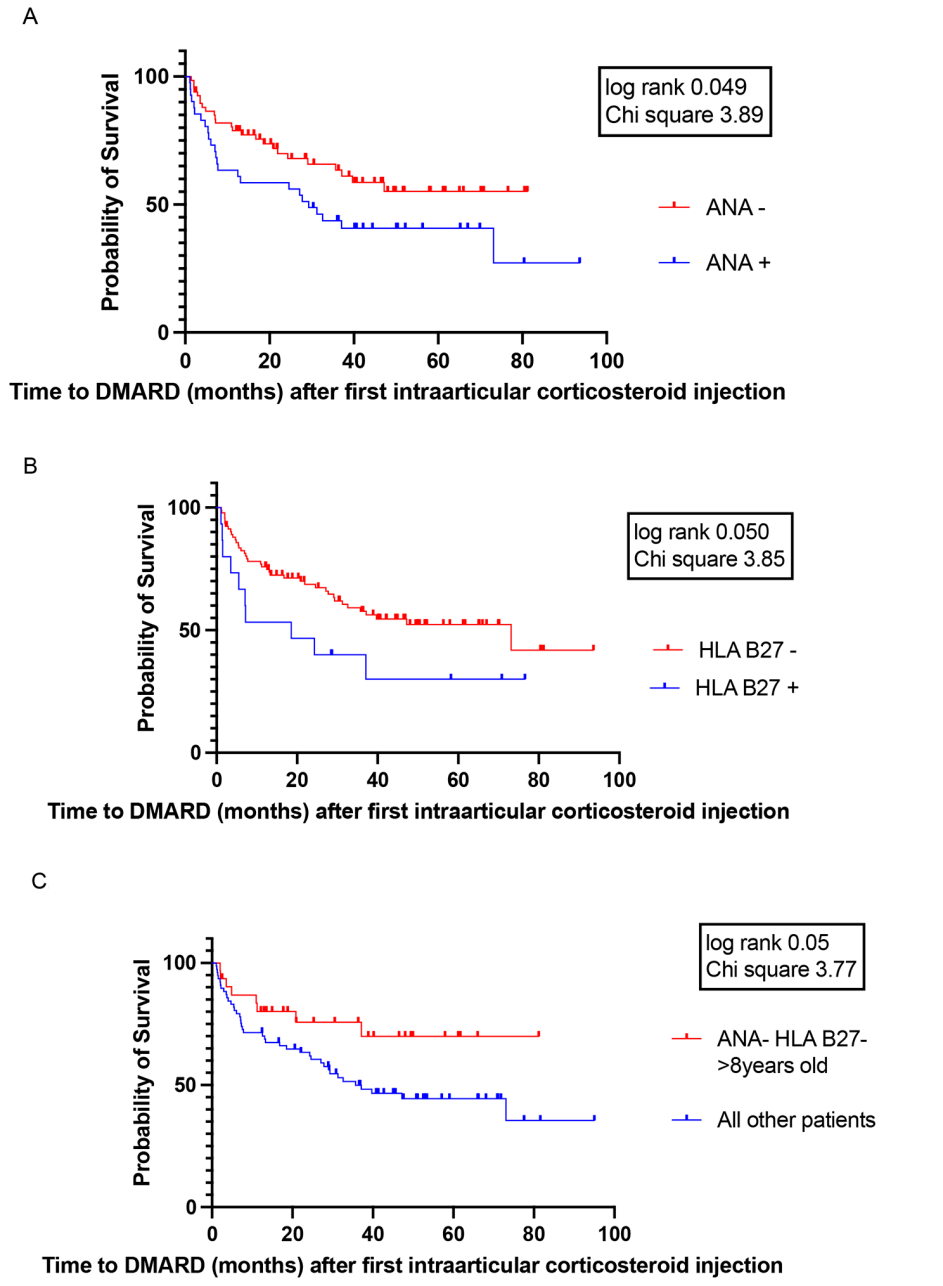
Survival functions from Kaplan Meier curves, showing difference in time to DMARD after first intra-articular corticosteroid injection in selected subgroups. **A**. Survival functions from Kaplan Meier curves, showing difference in time up to the DMARD after first intra-articular corticosteroid injection in ANA positive and ANA negative patients. **B**. Survival function from Kaplan Meier curves, showing difference in time up to the DMARD after first intra-articular corticosteroid injection in patients with and without HLA B27 antigen. **C**. Survival function from Kaplan Meier curves, showing difference in time up to the DMARD after first intra-articular corticosteroid injection in patients, that were older than 8 years at disease onset and were ANA and HLA B27 negative in comparison to all other patients

Of 38% of patients who were ANA positive, 73% (30/41) required further therapy, 12.2% (5/41) required only additional IAC, 61% (25/41) required MTX and 31.7% (13/41) required additional biological therapy. Using the log-rank test (Mantel-Cox) of survival analysis ANA positivity was associated with the need for systemic therapy (*P* = 0.049, chi square 3.89). Shown in Figs. 1B and 4A.

### HLA B27 antigen

Of the 14% of patients who were HLA B27 positive, 80% (12/15) required further therapy, 73.3% required systemic therapy (10 patients MTX, 1 patient SSZ) and 40% (6/15) required additional biological therapy. Using the log-rank test (Mantel-Cox) of survival analysis, HLA B27 antigen was associated with the need for systemic therapy (*P* = 0.050, chi square 3.85). Shown in Figs. 1C and 4B.

In the subgroup of patients, that were ANA negative, HLA B27 negative and older than 8 years at the time of first IAC, only 25.8% (8/31) required systemic and only 9.6% (3/31) required biologic therapy. Using the log-rank test (Mantel-Cox) of survival analysis these patients had less chance to need systemic therapy in the course of their disease (*p* = 0.050, chi square 3.77). Shown in Fig. 4C.

### Uveitis

6% (7/109) of patients developed uveitis in the course of disease, 57.1% (4/7) of these patients were ANA positive, 42.3% (3/7) of these patients were HLA B27 positive, one patient with uveitis was ANA and HLA B27 positive. Using the log-rank test (Mantel-Cox) of survival analysis ANA positivity was not significantly associated with uveitis (*p* = 0.38, Chi square 0.75). Uveitis was first noted average 25.4 months (2.5–78 months) after IAC. All but one patients with uveitis required systemic therapy.

### Trauma

Four patients had minor trauma in the history before onset of arthritis. Imaging did not show any traumatic injury to the anatomical structures. Three of these did not require any further therapy and one required biologic therapy.

### Orthopedic conditions

One patient had in the follow-up hondromalatia patelae and meniscus pathology and required orthopedic intervention. The patient had this pathology after being treated with MTX and biologic drug for extended oligoarthritis already for 3.5 years. One patient had non ossifying fibroma on a site distant from the site of arthritis.

### Other accompanying disorders

Two patients had thyroid disease, one patient had vitiligo, there were no other autoimmune diseases. One patient was treated for Hodgkin lymphoma and finished treatment 2 years before arthritis onset. One patient had persistent thrombocytopenia, one patient had phenilketonuria. One patient had epilepsy, one patient was autistic. Two patients were treated for suspected infection with Borrelia burgdorferi prior to IAC. Both were treated with antibiotic before the negative serology excluded infection.

## Discussion

To our knowledge this is the first study observing the children with juvenile idiopathic oligoarthritis from the view of IAC as the first treatment. The ILAR classification is not ideal, in the 17 year follow-up study 44% of patients changed disease subtype in the course of the disease [5]. In 2019 a new classification was proposed to distinguish forms of chronic arthritis that are typically seen in children from those that represent the childhood counterpart of diseases observed in adults [8]. The criteria have not been validated. The approach to treatment is therefore the most important when the patients is seen for the first time. For that reason we used the term juvenile idiopathic oligoarthritis as used in the treatment guidelines [6]. Most studies and larger databases focus only on patients after they already require systemic therapy, whereas the first treatment for oligoarthritis is IAC [6]. In our cohort more than half of the children with juvenile idiopathic oligoarthritis after first IAC did not require systemic therapy for arthritis and achieved long-term remission off therapy. This is valuable information for practicing clinician to be able to give this comforting information to the patient/parent. We also analysed the ILAR categories in the cohort and patients with persistent oligoarticular JIA required less systemic therapy than other categories, only in 35% patients, while all patients with extended oligoarticular JIA and 72.7% with ERA requred systemic therapy in the course of the disease. Nevertheless, JIA is a complex chronic immune mediated disease [9]. Other unrecognized factors, such as reactive arthritis, infections, orthopedic conditions or minor trauma might be influencing the development of arthritis in this subgroup that over years proved to be transient. We did not fully confirm this though, as there were only 5 patients reporting minor trauma prior to arthritis and only 2 patients had orthopedic conditions. Moreover, some children required additional treatment for arthritis years after being in remission after first IAC, delineating the fact, that immunologic routes are still inclined towards joint inflammation when exposed to the right trigger. Still, it would be important to consider this group of patients in a future classification. Similarly, a distinct clinical entity persistent monoarticular JIA has recently been proposed and analysed by an Italian group and suggested as a subgroup with specific characteristics [10]. One of the most distinct characteristics in monoarticular JIA was benign joint hypermobility, suggested as a contributing factor to more local mechanical nature [10]. Benign joint hypermobility was not evaluated in our study. The number of injected joints was not associated with the requirement for systemic treatment but it was a strong predictor for the need for biologic therapy later in the course of the disease, which is in line with previous studies [1, 11, 12]. 

Our cohort is a reliable representation of a real-world clinical group of patients with juvenile idiopathic oligoarthritis, as we are the only tertiary pediatric rheumatology centre in the country and all consecutive patients were enrolled. The cohort is well defined, the demographics are in line with previous data and also representative of the oligoarthritis group of our published JIA cohort [13]. In the historical cohorts the remission rates of oligoarthritis after 6–10 years from disease onset range from 23 to 47% [1]. The summary of 21 outcome studies published between 1995 and 2003 showed long-term remission rates in oligoarticular JIA between 36 and 84% [11]. Nevertheless, in these studies there was no data on treatment. Treatment in rheumatology has made an immense progress in the last decades. Our cohort is more recent, with children developing disease earliest in 2015 and at last follow up visit 89.7% had inactive disease in the whole cohort and 77% in those that required systemic therapy, which is the reflection of the biologic era and treat-to-target approach [14]. 

In our study, early age at presentation was an important predictor for systemic therapy. In a Spanish study on then already adult JIA patients, younger age at disease onset was predictive of higher disability in adult life [15]. In a French cohort of 207 children with oligoarticular JIA with a very young mean age at onset – 3.9 years, 50% developed a polyarticular course, which also hints that early onset oligo-JIA is inclined towards extension and therefore requirement of more than just local therapy [12]. 

### ANA positivity

In a 17-year follow up study of children with JIA, ANA positivity at baseline was associated with disease activity duration [5]. In a systematic literature review of early predictors of JIA outcome, ANA was not associated with the outcome [16]. The review made a joint analyses for all JIA categories together and it seems the correlation might only exist for the oligoarticular disease. In our study, presence of ANA was strongly associated with the need for systemic therapy. An important clinical implication is to follow the ANA positive patients after IAC more carefully.

### HLA B27 antigen

The presence of HLA B27 antigen proved to be a strong indicator for the future need for systemic and biologic therapy. The prevalence of HLA B27 in general population varies depending on the ethnic and geographic factors. It is commonly found in individuals with Caucasian descent where it is estimated to be present in approximately 8% of individuals and even more commonly in the Nordic countries. It is less common in other populations [17]. All our patients were White and the incidence of HLA B27 in our cohort was 13.1%, which is as expected higher than in the general population. In the Nordic cohort of JIA patients the prevalence of HLA B27 was 21%, probably because of a known higher background prevalence [18]. This near population based cohort study with 8 year follow-up also showed that the presence of HLA B27 was associated with higher odds of not being in remission off therapy after 8 year follow up, and 44% of HLA 27 positive JIA patients required systemic therapy, and 20% later required biologic therapy [18]. This is very consistent with our findings, suggesting that HLA B27 should be a part of routine laboratory investigation in all JIA patients, not only in diagnosing arthritis with enthesitis. Moreover, patients with HLAB27 antigen after IAC should be followed more carefully. An older study, conducted before the age of biologics associated presence of HLA B27 with more aggressive course and worse outcome in patients with JIA [19].

The best prognosis in our cohort had the patients, who were both ANA and HLA B27 negative and developed arthritis after 8 years of age. Out of them, only one quarter required systemic therapy and less than 10% required further biologic therapy.

### Uveitis

The data from the largest cohorts show that the incidence of uveitis in JIA ranges from 10 to 25% [20–22]. In our study it was 10%. In acohort of more than 1000 JIA patients, 75% of those who developed uveitis over the course of the study had it diagnosed within 3.2 years of the date of diagnosis of JIA, with a mean time to diagnosis of uveitis of 1.8 years [20]. This is in concordance with our data, where uveitis was first noted in average 2 years after first IAC. The mean follow up of more than 4 years in our study suggests that not many children are expected to develop uveitis afterwards and the low prevalence might be associated to early systemic treatment in the first years of the disease. Uveitis is more common in ANA positive patients [20, 21]. Our study confirmed this, as 53% patients with uveitis were ANA positive in comparison to 37% of the patients, who did not develop uveitis. The difference did not reach statistical significance due to small number of patients with uveitis.

The limitations of our study are the retrospective nature and size of the sample.

## Conclusion

Almost 40% of children with juvenile idiopathic oligoarthritis requiring IAC as first treatment did not need any further therapy or develop chronic rheumatic disease. Younger age at presentation, ANA positivity and presence of HLA B27 were important predictors for those that required systemic therapy, number of injected joints was an important predictor for future need for biologic therapy, indicating more careful follow-up in these patients. Children with the best prognosis were older than 8 years at presentation and were ANA and HLA B27 negative.

## Data Availability

No datasets were generated or analysed during the current study.

## Abbreviations

ACR: American College of Rheumatology
ANA: Anti-nuclear antibodies
BB: Borrelia burgdorferi
bDMARD: Biologic disease modifying anti-rheumatic drug
cDMARD: Conventional disease modifying antirheumatic drugs
IAC: Intra-articular injection of corticosteroid
JIA: Juvenile idiopathic arthritis
ILAR: International League of Associations for Rheumatology
ERA: Enthesitis related arthritis
MTX: Methotrexate
UCHL: University Children’s Hospital (UCHL), Ljubljana
